# African *Salmonella enterica* serovar Typhimurium ST313 isolates prevent reactive oxygen species production by human neutrophils via elevated PgtE expression

**DOI:** 10.1128/mbio.01609-25

**Published:** 2025-07-14

**Authors:** Annica R. Stull-Lane, Lizbeth Camacho, Amber E. R. Van Hecke, Blanca Perez-Sepulveda, Eli J. Bejarano, Maria G. Winter, Sebastian E. Winter, Jay C. D. Hinton, Andreas J. Bäumler, Hirotaka Hiyoshi, Renée M. Tsolis

**Affiliations:** 1 Department of Medical Microbiology and Immunology, University of California at Davis 8789, Davis, California, USA; 2 Institute of Infection, Veterinary and Ecological Sciences, University of Liverpool 105724 , Liverpool, England, United Kingdom; 3 Division of Infectious Diseases, School of Medicine, University of California at Davis 12218, Davis, California, USA; 4 Department of Bacteriology, Institute of Tropical Medicine, Nagasaki University 196838, Nagasaki, Japan; University of Utah, Salt Lake City, Utah, USA

**Keywords:** *Salmonella*, neutrophils, complement

## Abstract

A recently emerged *Salmonella enterica* serotype Typhimurium variant, ST313, is a frequent cause of bloodstream infections associated with high mortality rates in sub-Saharan Africa. While the interaction of ST313 strains with macrophages has been studied extensively, interactions with other innate immune effectors such as neutrophils have been largely ignored. We found that a subset of ST313 strains induces an attenuated neutrophil oxidative burst, which can facilitate increased bacterial survival within human neutrophils. The ST313 isolates that avoid triggering potent reactive oxygen species production by neutrophils express elevated levels of the protease PgtE. Increased *pgtE* expression promotes decreased complement deposition on *S*. Typhimurium and a concomitant increase in disseminated infection in mice. Our results show that high-level PgtE expression contributes to evasion of the neutrophil oxidative burst by *S*. Typhimurium, suggesting a possible mechanism contributing to invasive *S*. Typhimurium disease.

**IMPORTANCE:**

The *S. enterica* serotype Typhimurium omptin family protease PgtE has long been known to cleave complement factors bound to the bacterial surface, but a role for complement inactivation by PgtE in avoidance of serum bactericidal activity was found only for bacteria having rough lipopolysaccharide. Here we show that PgtE-mediated interference with the complement cascade helps evade the oxidative burst of human neutrophils. Our results suggest a mechanism by which increased expression of this protease by *S*. Typhimurium strains associated with severe bloodstream infection promotes evasion of the innate immune response by *S*. Typhimurium.

## INTRODUCTION

Non-typhoidal *Salmonella* (NTS) infections are widely associated with gastroenteritis linked to the industrialization of food production, being one of the main causes of foodborne outbreaks with over 65,000 confirmed cases in Europe in 2022 (1). NTS infections represent a significant public health problem worldwide, of which the main isolated serovars are Enteritidis and Typhimurium. However, new lineages of invasive non-typhoidal *Salmonella* (iNTS) pose a greater threat to human health, causing about 77,000 deaths each year due to bloodstream infection, mostly in sub-Saharan Africa (2). A key factor associated with the prevalence of iNTS bloodstream infections in sub-Saharan Africa is the emergence of distinct, antibiotic-resistant lineages of *S*. Typhimurium sequence type (ST) 313 (ST313) and of two epidemic clades of *S*. Enteritidis (West African and Central/East African clades) (3, 4). The *S*. Typhimurium ST313 lineages L1, L2.0, L2.1, and L2.2 are associated with a higher ratio of bloodstream to gastrointestinal human isolates than the *S*. Typhimurium ST19 lineage, which is predominant in Europe and North America. However, the genetic basis underlying the increased propensity of the ST313 lineage to cause invasive bloodstream infection remains incompletely understood. Genomic epidemiology studies comparing African isolates with isolates in industrialized countries have revealed genomic differences involving accessory genome and pseudogenes in the African isolates, such as single-nucleotide polymorphisms (SNPs) and insertion sequences that have been predicted to cause inactivation of multiple genes involved in interaction with the host (5). Strikingly, some of these small genomic changes result in altered interactions with the innate immune system in animal models and increased dissemination from the intestine (6–10).

During both gastroenteritis and invasive disease, neutrophils are crucial in controlling *S*. Typhimurium infection (11). In the context of inflammatory diarrhea, neutrophils are recruited to the lamina propria and translocate to the gut lumen to engulf *Salmonella* (12). During invasive infection, *Salmonella* disseminates from the gut to the lymphatics and bloodstream. Neutrophils from the bone marrow (BM) respond to infection at systemic sites like the liver, spleen, and blood (11). The work of multiple groups supports the idea that neutrophils work cooperatively with macrophages to control *Salmonella* infection at systemic sites: studies in mice have shown that while macrophages take up *Salmonella*, death of infected macrophages recruits neutrophils, which are important for killing extracellular bacteria (13, 14). Furthermore, a population of bacteria disseminating from the intestine via efferent lymphatics has been shown to be extracellular (15).

Underlying conditions that predispose to iNTS disease include concurrent and recent malaria, malnutrition in children, and HIV in adults; all these examples involve either neutropenia or generation of non-functional neutrophils (16–20). Notably, neutropenic patients such as those undergoing cancer chemotherapy are more susceptible to *Salmonella* bacteremia (21), another illustration of the importance of *Salmonella*-neutrophil interactions in controlling disseminated infections. Mouse models have demonstrated that the generation of dysfunctional neutrophils in malaria and vitamin A deficiency plays a causal role in enhanced susceptibility to *S*. Typhimurium (22, 23). However, how the genomic differences of ST313 lineages affect interactions of *S*. Typhimurium with neutrophils during disseminated infection is not known. In this study, we examined differences between a set of African ST313 *S*. Typhimurium strains and ST19 strains from other global sites in their interaction with neutrophils, drawing on evidence from primary human neutrophils *ex vivo*, a human neutrophil-like cell line *in vitro*, mouse neutrophils *ex vivo*, and a murine infection model.

## RESULTS

### An African *S*. Typhimurium isolate elicits lower levels of reactive oxygen species from neutrophils

**Fig 1:**
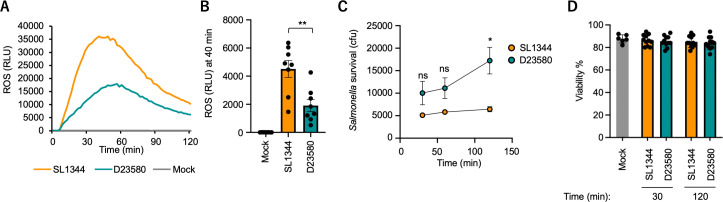
An African *S*. Typhimurium strain elicits lower levels of bactericidal reactive oxygen species from neutrophils. Human HL-60 cells were infected with the indicated *S*. Typhimurium strains at a multiplicity of infection of 10 bacteria per neutrophil and incubated for 2 h. ROS production was monitored by chemiluminescence. (**A**) Representative experiment showing generation of ROS over time. (**B**) Average chemiluminescence from eight independent experiments, 40 min after addition of bacteria. Bars represent means ± SE. Each dot represents the mean of samples from a single experiment. Bars represent means ± SE. Differences between groups were determined using a Mann-Whitney test. **, *P* < 0.01. (**C**) Recovery of *S*. Typhimurium from 5 × 10^4^ neutrophils at the indicated time points (*n* = 4). Statistical comparisons were made between SL1344 and D23580 within a single time point. CFU, colony-forming unit; mock, mock infection; ns, not significant; RLU, relative luminescence unit. Differences between groups were determined using a two-way analysis of variance test with the Geisser-Greenhouse correction and a post hoc Šidák multiple comparison test performed on log-transformed values. *, *P* < 0.05; ** *P* < 0.01. (D) Quantification of HL-60 cell viability before inoculation (Mock) and 30 and 120 min after *S.* Typhimurium inoculation at a MOI of 10, using Trypan Blue exclusion. Viability is expressed as % of cells that were negative for Trypan Blue staining. Data are combined from three independent experiments.

While studying the interaction of different *S*. Typhimurium strains with neutrophils, we noticed a striking difference in the human neutrophil response to the gastroenteritis-associated ST19 strain SL1344 and the invasive Malawian ST313 isolate D23580. For these experiments, HL-60 cells were differentiated and inoculated with either SL1344 or D23580, and the generation of reactive oxygen species (ROS) was followed for 2 h after infection with chemiluminescence. Over the 2 h time course, we observed that the ROS response to SL1344 peaked between 30 and 60 min and decreased thereafter (Fig. 1A), while the response to D23580 was approximately one-half of that detected for SL1344 (Fig. 1A and B). Consistent with ROS being an important killing mechanism of neutrophils against *Salmonella*, D23580 increased in numbers within neutrophils between 60 and 120 min, while recovery of intracellular SL1344 remained constant and was significantly lower than D23580 at 120 min, suggesting the possibility that reduced ROS production may open an intracellular replication niche within neutrophils for D23580 (Fig. 1C). At 30 min post-infection, no significant difference between cell-associated D23580 and SL1344 was found, suggesting that while uptake of D23580 may be increased slightly compared to SL1344, it does not appear to be the major driver of its increased intracellular survival compared to SL1344 (*P* > 0.05, Fig. 1C).

### Reduced neutrophil ROS responses to an ST313 isolate are conserved among individuals

**Fig 2:**
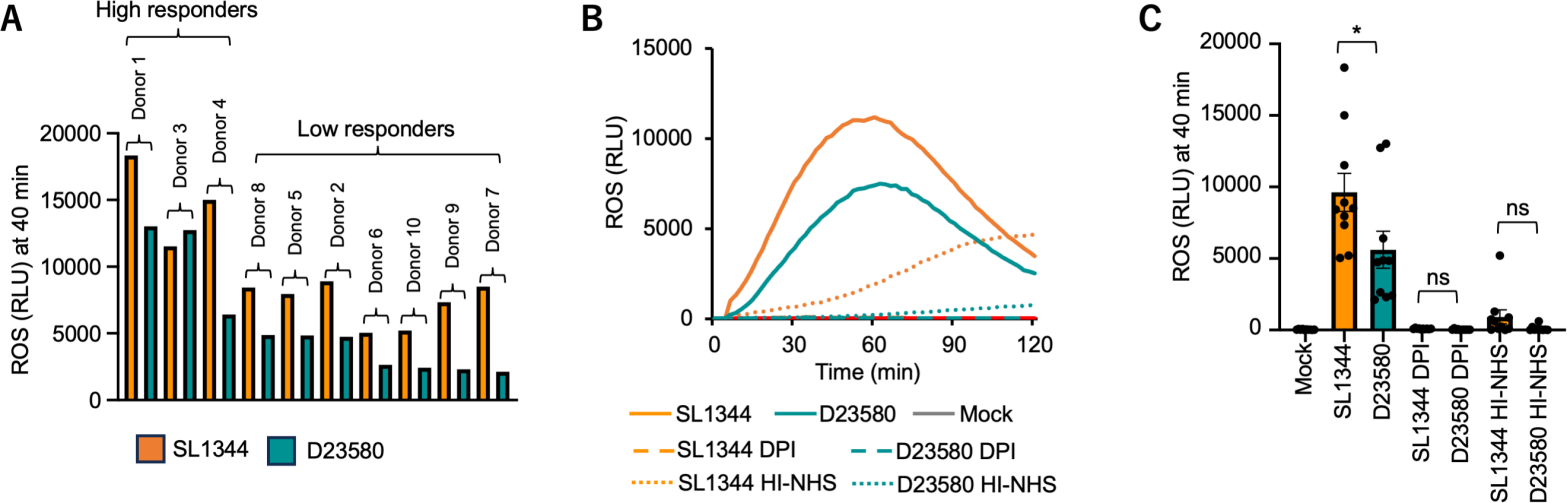
Reduced neutrophil ROS responses to D23580 are conserved among individuals and involve factors present in serum. (A) Neutrophils isolated from blood of human donors were infected with *S*. Typhimurium strains at a multiplicity of infection of 10, and ROS production was monitored by chemiluminescence. (**B**) Production of ROS over time by *S*. Typhimurium-infected neutrophils from donors (*n* = 10) incubated with autologous serum that was untreated or heat-inactivated. Neutrophil cultures were pre-treated with vehicle alone or with diphenyleneiodonium (DPI) to inhibit the oxidative burst. (**C**) Average chemiluminescence from five independent experiments, 40 min after addition of bacteria. Each dot represents the mean of samples from a single experiment. Bars represent means ± SE. HI-NHS, heat-inactivated normal human serum; mock, mock infection; ns, not significant; RLU, relative luminescence unit, ROS, reactive oxygen species. Differences between groups were determined using an unpaired *t*-test. *, *P* < 0.05.

To investigate the basis for the reduced neutrophil response to D23580, we used human primary blood neutrophils (*n* = 10 donors). The donors segregated into two groups, according to the magnitude of their ROS response to *S*. Typhimurium. In 9 of 10 donors, the neutrophil ROS response to SL1344 was higher than the response to D23580 (Fig. 2A). While some inter-individual variation between donors in the kinetics of the ROS response was observed, on average, the peak ROS production was observed at a later time point in primary neutrophils (60–70 min) than in the HL-60 cells, which may reflect the different origins of the cells. The chemiluminescent response could be inhibited by diphenyleneiodonium (DPI), an inhibitor of NADPH oxidase, showing that NADPH oxidase is responsible for the ROS measured. Furthermore, most of the immediate ROS response was dependent on heat-labile serum components, since heat inactivation of serum used to opsonize *S*. Typhimurium reduced and delayed the onset of the ROS response (Fig. 2B and C).

**Fig 3:**
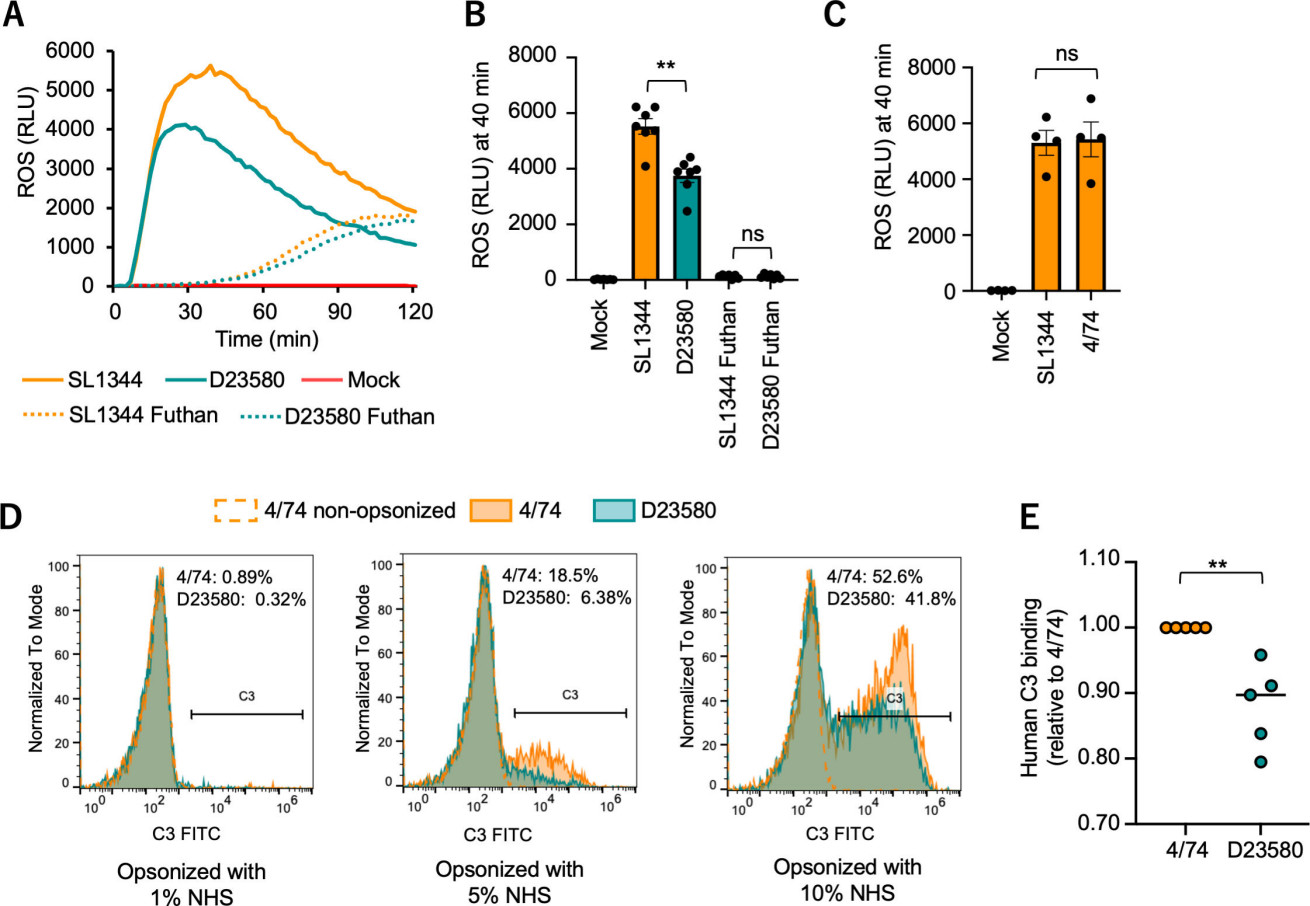
Induction of neutrophil ROS by *S*. Typhimurium depends on complement. (A) Production of ROS over time by *S*. Typhimurium-infected HL-60 cells incubated with normal human serum or with the complement inhibitor Futhan. Data shown are combined from seven independent experiments. (**B**) Average chemiluminescence from seven independent experiments, 40 min after addition of bacteria at a multiplicity of infection of 10. (**C**) 4/74 and its laboratory derivative SL1344 elicit identical levels of ROS from neutrophilic HL-60 cells. Data shown are from four independent experiments. (**D**) Quantification of complement factor C3 binding on the surface of *S*. Typhimurium after opsonization with 1%, 5%, or 10% of human serum. Shown is a representative experiment, with C3-positive events designated by a horizontal line. Percentage of C3-positive bacteria for each strain is given in the upper right of each plot. (**E**) Average relative C3 binding on the surface of *S*. Typhimurium after opsonization with 10% serum was measured by flow cytometry. Data shown are from five independent experiments. Each dot represents the mean of technical replicates from a single experiment. Mock, mock infection; NHS, normal human serum; ns, not significant; RLU, relative luminescence unit. Differences between groups were determined using a Mann-Whitney test. **, *P* < 0.01.

Since complement factors, especially C5a, play a role in priming the oxidative burst (24), we first asked whether the different ROS levels elicited by ST313 and ST19 strains could be related to differential complement activation by the two strains. The complement inhibitor nafamostat mesylate, also known as 6-amidino-2-naphthyl p-guanidinobenzoate dimethanesulfonate (Futhan), reduced and delayed the ROS response to *S*. Typhimurium. Notably, treatment with Futhan abolished the difference in ROS response to SL1344 and D23580, suggesting that a factor contributing to the reduced neutrophil ROS response to D23580 may be complement binding (Fig. 3A and B). To further test this idea of differential interactions of complement and pathogen strain, we measured complement C3 binding to the surface of *S*. Typhimurium, since recruitment of C3 to the bacterial surface is a feature of classical, lectin, and alternative pathways of complement activation. For these experiments, we used the parent strain of SL1344, 4/74, which differs from SL1344 by eight SNPs (25) that do not affect neutrophil ROS generation in response to infection (Fig. 3C). C3 binding to the surface of 4/74 and D23580 was quantified by flow cytometry, after opsonization of bacteria with human serum. Opsonization with 5% human serum demonstrated a higher proportion of 4/74 bacteria (18.5%) than D23580 (6.38%) associated with complement C3 (Fig. 3D and E). These results are consistent with a previous report of reduced complement deposition on D23580 as compared to 4/74 (7) and further suggest that the lower ROS induction elicited by D23580 upon encountering neutrophils results from a reduction in complement activation.

### Neutrophil ROS production in response to *S*. Typhimurium infection correlates with the C/T promoter polymorphism at the −10 region of *pgtE*

**Fig 4:**
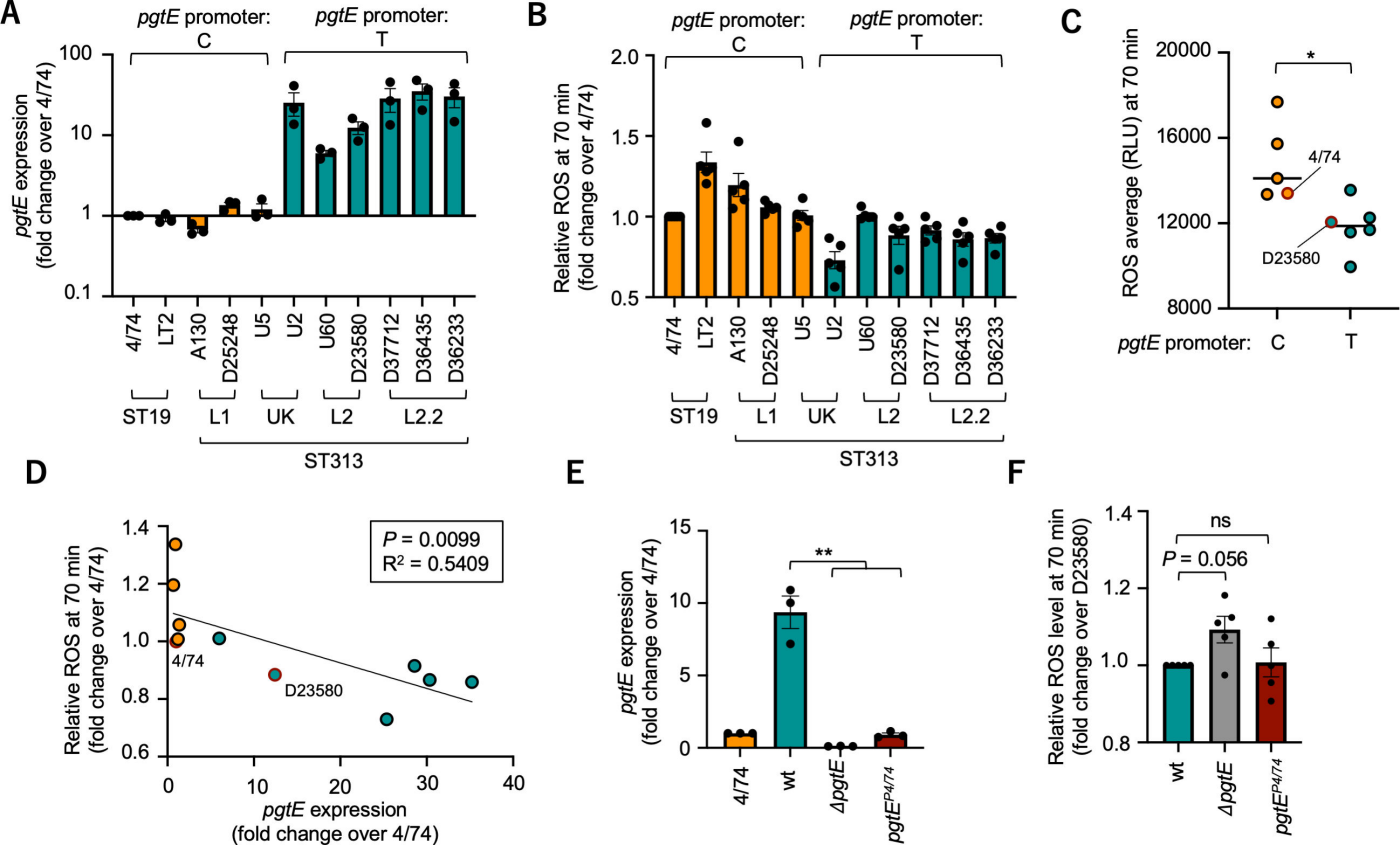
*S*. Typhimurium isolates with higher *pgtE* expression elicit less ROS on encounter with human neutrophils. (A) *pgtE* expression was compared between groups of ST19 strains and different lineages of ST313 strains by RT-qPCR in three independent experiments. Bars represent geometric mean ± SE. (**B**) Comparison of ROS levels at 70 min after infection of primary human neutrophils at a multiplicity of infection of 10 in five independent experiments. Each dot represents the mean ROS measurement from a single experiment, and bars represent means ± SE. (**C**) Comparison of mean ROS levels in panel **B**, grouped by *pgtE* promoter single-nucleotide polymorphism. (**D**) Correlation between ROS levels and *pgtE* expression level in individual *S*. Typhimurium strains. (**E**) Expression of *pgtE* by *S*. Typhimurium strains carrying different alleles of *pgtE*. Transcript abundance was determined by RT-qPCR. Dots represent means of individual experiments (*n* = 3), and bars represent means ± SE. (**F**) Comparison of ROS levels in human neutrophils infected with the indicated *S*. Typhimurium strains. Dots represent means of individual experiments (*n* = 5), and bars represent means ± SE. RLU, relative luminescence units; ROS, reactive oxygen species; wt, wild type. Differences between groups were determined using a Mann-Whitney test. *, *P* < 0.05; **, *P* < 0.01.

Among the genomic differences between D23580 and SL1344 that could affect complement activation is a recently identified promoter polymorphism in *pgtE*, which encodes a cell envelope protein of *S*. Typhimurium that belongs to the omptin family of cell envelope proteases (25). It was reported by Hammarlöf et al. that a subset of ST313 strains belonging to lineage 2 contains an SNP (C/T) in the −10 promoter region of *pgtE*, leading to increased *pgtE* expression (7). Interestingly, this polymorphism caused an increased ability to cleave multiple complement factor components, including C3, C3b, C4b, C5, B, and H (7, 26, 27). Given the role of complement in stimulating the neutrophil oxidative burst, we therefore asked whether this SNP-based variation in *pgtE* expression could contribute to a reduction in neutrophil ROS after infection with ST313 strains. For these experiments, we utilized a panel of sequenced isolates representative of ST19 or ST313 lineages 1, UK-ST313, 2 or 2.2 (5). A phylogenetic tree indicating the relatedness between the ST313 lineages, and which isolate belongs to which lineage, is shown in Fig. S1. As reported previously (7), ST19 strains as well as ST313 strains from lineage 1 and one of the UK lineage ST313 strains that carry the *pgtE* T promoter polymorphism expressed higher levels of *pgtE*, and we were able to replicate this finding by reverse transcriptase-quantitative polymerase chain reaction (RT-qPCR) (Fig. 4A). Strains with the T polymorphism also elicited lower ROS responses from human blood neutrophils (Fig. 4B and C; Fig. S2). Furthermore, we observed a negative correlation between *pgtE* expression level of *S*. Typhimurium strains and the magnitude of ROS production elicited from human blood neutrophils (Fig. 4D), suggesting that elevated *pgtE* expression levels in ST313 strains, via a reduction in complement activation, may lower the ROS response to *S*. Typhimurium on contact with neutrophils.

### Differences in *pgtE* expression partially account for the differences in ROS production elicited by *S*. Typhimurium in human neutrophils

To determine whether differences in *pgtE* expression between 4/74 and D23580 are sufficient to recapitulate the reduced ROS response of neutrophils to *S*. Typhimurium infection, we examined the ROS responses to wild-type D23580, an isogenic mutant of D23580 with a deletion of *pgtE*, and a D23580 mutant in which the promoter of *pgtE* was mutated to the C variant found in 4/74 (C^4/74^) (Fig. 4E). Under *in vitro* growth conditions used to grow *S*. Typhimurium for neutrophil infection assays, *pgtE* transcripts were 10-fold more abundant in D23580 compared to 4/74. The D23580 strain carrying the C^4/74^ promoter variant expressed *pgtE* at a level similar to 4/74, confirming previous reports (7) (Fig. 4E). After infection of neutrophils, the D23580 *pgtE* deletion mutant elicited a trend (*P =* 0.056) toward increased ROS production from human blood neutrophils (Fig. 4F). However, the C^4/74^ promoter variant did not consistently increase neutrophil ROS to the same extent as abrogation of *pgtE* expression, suggesting that while this promoter variant may contribute to the observed differences in ROS production between the different *S.* Typhimurium strains, it is not sufficient to mediate the phenotype.

### Comparative analysis with mouse neutrophils *ex vivo* and an *in vivo* mouse infection model demonstrates a conserved phenotypic difference in ROS induction and *pgtE*-dependent survival of *S.* Typhimurium strains

**Fig 5:**
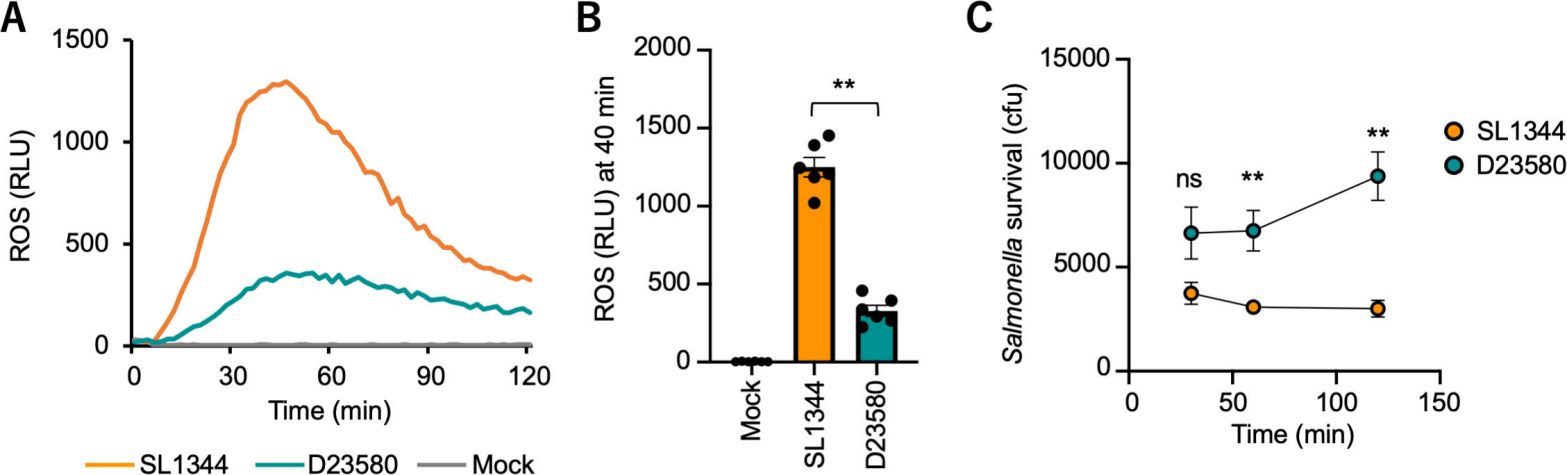
The reduction in neutrophil ROS elicited by D23580 and its ability to replicate with neutrophils are conserved in mice. (A) Representative experiment showing generation of ROS by bone marrow-derived neutrophils from C57BL/6J mice after incubation with *S.* Typhimurium at a multiplicity of infection of 10. (**B**) Average chemiluminescence from six independent experiments, 40 min after addition of bacteria. Each dot represents the mean of samples from a single experiment. Bars represent means ± SE. Differences between groups were determined using a Mann-Whitney test. **, *P* < 0.01. (**C**) Recovery of S. Typhimurium from mouse primary neutrophils at the indicated time points (*n* = 6). Statistical comparison was made between SL1344 and D23580 within an individual time point, using a two-way analysis of variance test, with the Geisser-Greenhouse correction and a post-hoc Šidák multiple comparison performed on log-transformed values. **, *P* < 0.01. CFU, colony-forming unit; Mock, mock infection; ns, not significant; RLU, relative luminescence unit; ROS, reactive oxygen species.

Given the clinical importance of disseminated *S.* Typhimurium infection caused by isolates of the ST313 lineages in sub-Saharan Africa, we sought to determine the importance of *pgtE* expression *in vivo* using mice. First, we confirmed that the reduction in neutrophil ROS response elicited by D23580 as compared to SL1344 was conserved between humans and mice by comparing the response of neutrophils enriched from mouse bone marrow to infection with *S.* Typhimurium strains *ex vivo* (Fig. 5A and B). The overall ROS levels elicited by *S.* Typhimurium in murine neutrophils were lower than either HL-60 or primary human neutrophils (Fig. 1A and B). Multiple factors may contribute to this finding, including the mixed maturation stages of neutrophils isolated from bone marrow (23) and the reduced stability of serum complement factors in mice compared to humans. Furthermore, the ROS response in the murine neutrophils peaked around 40 min, which was most similar to the response in HL-60 cells (Fig. 1A and B). However, the trend for lower ROS generation in neutrophils inoculated with D23580 compared to SL1344 was consistent between all three *in vitro* neutrophil models. Furthermore, recovery of D23580 from mouse neutrophils increased over time, while SL1344 recovery remained constant over the 120 min time course of the experiment (Fig. 5C).

**Fig 6:**
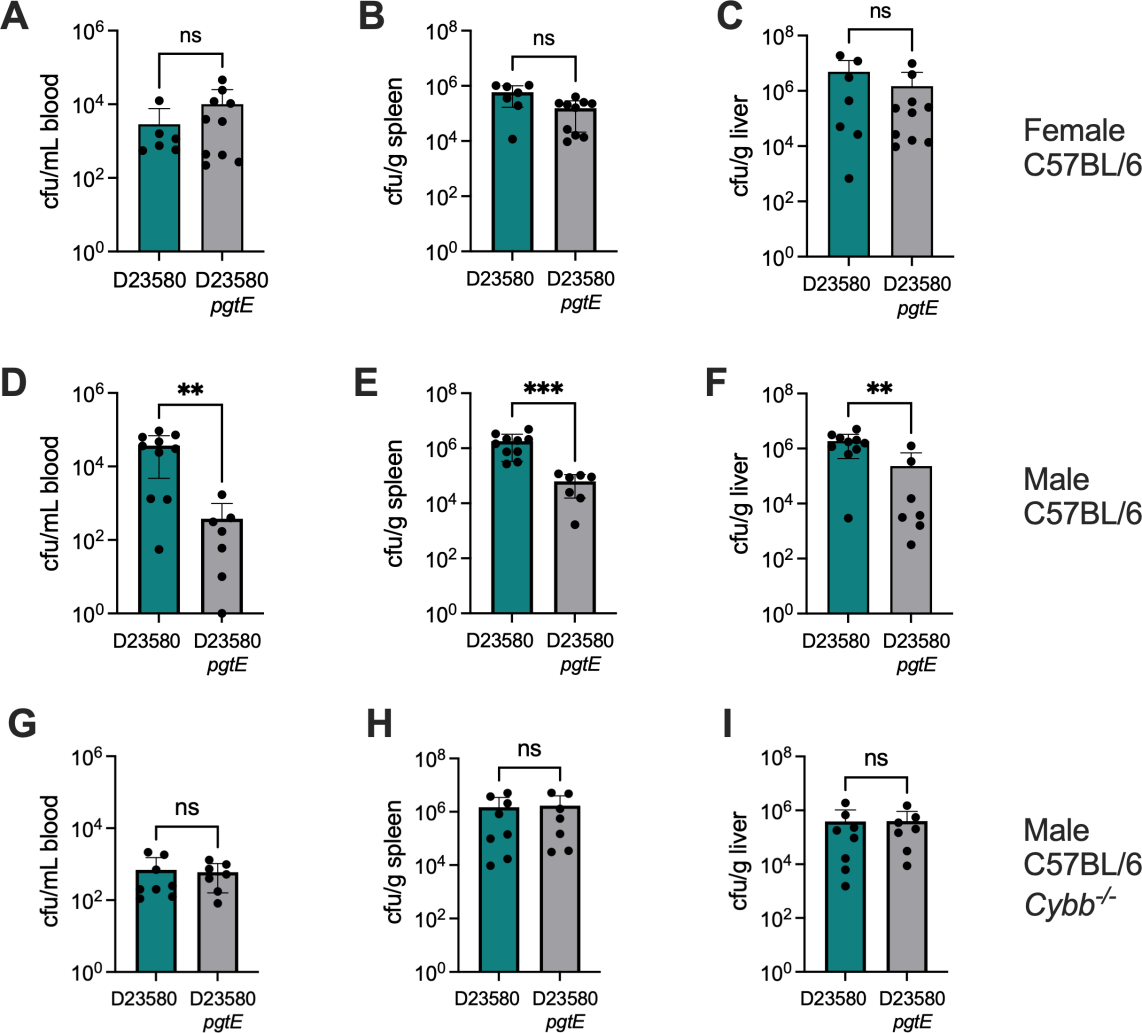
Expression of *pgtE* by *S*. Typhimurium D23580 increases systemic infection in male mice with a functional NADPH oxidase. Contribution of *pgtE* to infection of blood, liver, and spleen after infection with *S*. Typhimurium D23580 strains in female (**A–C**) and male (**D–F**) C57BL/6 mice or in congenic male *Cybb^−/−^* lacking a functional NADPH oxidase (G–I). Colonization was assessed at 24 h after inoculation via the IP route with 2 × 10^4^ CFU of the indicated strain. CFU, colony-forming unit; ns, not significant; wt, wild-type D23580. Significance of differences between groups was determined using a Mann-Whitney test. **, *P* < 0.01; ***, *P* < 0.001.

Based on these results, we inoculated mice via the intraperitoneal (IP) route with D23580 or its isogenic *pgtE* deletion mutant and quantified bacterial loads in the spleen, liver, and blood. At 1 day post-infection, we observed no effect of PgtE deficiency on systemic colonization of female mice by *S.* Typhimurium D23580 (Fig. 6A through C). In contrast, in male mice, D23580 (wild type [wt]) was present in 10- to 100-fold higher numbers than the *pgtE* mutant in the spleen, liver, and blood (Fig. 6D through F). Consistent with the role we identified for PgtE in evasion of ROS-mediated killing, the *pgtE* phenotype was absent in male *Cybb*^−/−^ mice lacking the gp91 subunit of NADPH oxidase (Fig. 6G through I). These results show that the role of PgtE in preventing activation of the oxidative burst in neutrophils is also important in promoting disseminated *S.* Typhimurium infection in mice. The sex-specific phenotype of PgtE deficiency in mice is consistent with previously reported effects of sex hormones on neutrophil function, with estrogen driving higher antimicrobial activity of neutrophils in females than in males after sexual maturation (28). Taken together, our results show that PgtE-mediated evasion of neutrophil-mediated antimicrobial defenses contributes to the ability of *S.* Typhimurium to cause disseminated infection.

## DISCUSSION

Genomic analyses of the ST313 *S.* Typhimurium strains circulating in sub-Saharan Africa have provided a paradigm for how small genomic differences can drive phenotypic variation in disease outcomes. The ST313 strains have multiple phenotypic differences that may make them more likely to take advantage of underlying susceptibility factors prevalent in people living in sub-Saharan Africa. Here, we show that collectively, these genomic changes, particularly in lineage 2 isolates, affect the interaction of *S.* Typhimurium with neutrophils, a key component of the innate immune response that is essential for controlling disseminated infection. Previously, effects of genomic differences between ST19 and ST313 strains were identified in phenotypic variability in interactions with macrophages (9, 10, 29) or migratory dendritic cells (6), and this work extends our knowledge to interaction with neutrophils. Our focus on human neutrophils augments the translational potential of our findings in informing mechanistic targets for novel therapies.

One SNP located in the −10 promoter region of *pgtE*, *pgtE* T^D23580^, drives increased *pgtE* expression in lineage 2 and some UK lineage ST313 strains carrying the SNP. Increased PgtE expression has been shown to increase resistance of lipopolysaccharide (LPS)-rough *S.* Typhimurium to killing by human serum and to cause decreased C3b deposition on the cell surface (7). Our results show that ST313 strains carrying the T^D23580^ promoter SNP in *pgtE* can evade the oxidative burst of neutrophils, enabling them to survive and replicate within these phagocytes. Previous studies hypothesized that *pgtE* only played a role in LPS-rough *S.* Typhimurium, as no phenotype related to serum resistance, opsonophagocytosis, or macrophage survival could be found in bacteria with smooth LPS (26, 27, 30–32). Our results demonstrate a role for PgtE in the interaction of smooth bacteria with neutrophils by attenuating the neutrophil respiratory burst and enhancing neutrophil survival.

While the T^D23580^ promoter SNP in *pgtE* contributed to evasion of the oxidative burst, it only partially explains the differences between ST19 and ST313 strains in generating neutrophil ROS, suggesting that additional genomic differences between these lineages modulate the neutrophil oxidative burst. One example of variation in surface composition that could potentially alter neutrophil interaction could be the BTP1 prophage-encoded GtrC, which increases acetylation of LPS O-antigen (33, 34). Bacterial motility has also been implicated in stimulating the oxidative burst, suggesting that the reduced flagellin expression in ST313 strains (9, 35) could also be a factor in evasion of neutrophil oxidative killing.

In mice, we found a male-specific phenotype of attenuated disseminated disease with a *pgtE* deletion mutant, and this observation was dependent on functional NADPH oxidase. Epidemiologically, several studies across the globe have reported a male preponderance in the development of NTS bacteremia (36–39), which is consistent with known sex differences in immune responses related to hormonal differences and localization of several immune-related genes on the X chromosome (40–43). Transcriptional profiling of circulating neutrophils showed that human males, but not females, have a population of immature neutrophils that may be less able to kill bacteria such as *S.* Typhimurium (44). In mice, multi-omic profiling of neutrophils has provided support for the concept that male mouse neutrophils have a lower pro-inflammatory response than female mouse neutrophils (45). While more research is needed in the translational impact of comparative animal models, separating our mouse model findings by sex did help to illuminate a connection between *pgtE* and neutrophil function.

How could the small phenotypic differences in the induction of the bactericidal neutrophil oxidative burst identified in our study contribute to the ability of the ST313 strains to cause disseminated infections in specific regions globally? One answer may lie in the prevalence in sub-Saharan Africa of underlying conditions such as malaria, malnutrition, HIV, and sickle cell disease, all of which diminish the function of neutrophils, by preventing their maturation or diminishing their antimicrobial capacity (16, 22, 23, 46, 47). In children under 2, another group with increased susceptibility to invasive *Salmonella* disease, neutrophil antimicrobial capacity is reduced indirectly since young children have lower circulating anti-*Salmonella* IgG and IgM, which are important for fixing complement on the *Salmonella* surface to elicit an oxidative burst (48, 49). Thus, a small reduction in the ability to elicit bacterial ROS in the ST313 lineage 2 strains may, in hosts with already compromised neutrophil function, be able to increase the ability of *S.* Typhimurium to cause severe disseminated infections.

## MATERIALS AND METHODS

### Bacterial strains

The bacterial strains used in the present study are listed in Table 1. Bacterial cultures were routinely incubated with aeration at 37°C in Luria-Bertani (LB) broth (10 g tryptone and 5 g yeast extract supplemented with 5 g [Lennox] or 10 g [Miller] NaCl per liter) or on LB agar plates unless indicated otherwise. Antibiotics were added, as appropriate.

**TABLE 1:** Bacterial strains and primers for quantitative PCR (qPCR) used in this study

Materials	Relevant characteristics/genotype	Source/reference
*S*. Typhimurium ST19 strains		
4/74	ST19	(50)
SL1344	ST19, wild type derived from 4/74	(51)
LT2	ST19	(52)
SARA 1	Human *S.* Typhimurium isolate from Mexico	(53, 54)
SARA 6	Human *S.* Typhimurium isolate from the USA	(53, 54)
SARA 16	Human *S.* Typhimurium isolate from the USA	(53, 54)
SARA 19	Human *S.* Typhimurium isolate from Mexico	(53, 54)
*S*. Typhimurium ST313 strains		
A130	Lineage 1	(3)
D25248	Lineage 1	(55)
D26104	Lineage 1	(56)
D23580	Lineage 2, wild type	(3)
JH3813	D23580 pgtE, *∆pgtE::frt*	(7)
JH3812	D23580 *pgtE^P4/74^*, *P_pgtE_^4/74^*	(7)
U60	Lineage 2	(57)
D25352	Lineage 2	(56)
D24545	Lineage 2	(56)
D23005	Lineage 2	(56)
D37712	Lineage 2.2	(55)
D36435	Lineage 2.2	(55)
D36233	Lineage 2.2	(55)
U5	UK-ST313	(57)
U2	UK-ST313	(57)
Primers		
DH54	CGAACACTACATGCGCAAAC	(7)
DH55	ACCGCCCTTACCTTCTTCAT	(7)
qPCR_hns_f	TCTGAACAACATCCGTACTCTTC	(7)
qPCRhns_r	TTCTTCTTCACGACGCTCATTA	(7)

### Mouse lines

Female and male 7- to 8-week-old C57BL/6J (stock no. 000664) mice and *Cybb^tm1Din1^*/J mice (stock no. 002365) lacking the gp91phox subunit of NADPH oxidase were purchased from The Jackson Laboratories and bred at UC Davis. All animal experiments were approved by the Institution of Animal Care and Use Committee at the University of California, Davis. The exact number of mice used in each group is indicated in each graph or in the figure legend.

### Neutrophil isolation

Neutrophil experiments included assessments with primary human neutrophils *ex vivo*, cell line HL-60 cells *in vitro*, and/or primary mouse neutrophils *ex vivo*. Neutrophil viability was assessed by trypan blue exclusion. Primary human neutrophils used in this study were isolated from whole blood obtained from human volunteers using the EasySep Human Neutrophil Enrichment Kit (StemCell Technologies). HL-60 cells (ATCC CCL-240), a cell line derived from acute promyelocytic leukemia, were differentiated from promyelocytes into neutrophil-like cells by incubation in Roswell Park Memorial Institute (RPMI) 1640 medium (Thermo Fisher Scientific) containing 1.3% dimethyl sulfoxide (Sigma), 15% fetal bovine serum (FBS; Thermo Fisher Scientific), and 25 mM HEPES (Thermo Fisher Scientific) for 5 days, which resulted in a population of cells with mixed maturation stages ranging from myelocytes to segmented neutrophils (58). Primary murine neutrophils were isolated from the BM of murine femora using the EasySep Mouse Neutrophil Enrichment Kit (StemCell Technologies), which yields a population of cells with mixed maturation stages, based on Ly6G staining (23).

### ROS measurement assay

ROS production from HL-60 cells, primary human neutrophils, or primary mouse neutrophils was measured using chemiluminescence. Before infection, the neutrophils were treated with 5 µM NADPH-oxidase inhibitor (DPI chloride; Sigma-Aldrich, #D2926) for 30 min or with vehicle control. Neutrophils were washed and resuspended in phenol red-free RPMI 1640 (Thermo Fisher Scientific) containing 2% FBS, and luminol (Sigma, #123072) was added (final concentration, 1 mM). Approximately 5 × 10^4^ neutrophils in 90 µL were seeded into an opaque 96-well microplate (PerkinElmer), and basal luminescence was measured every 2 min for 5 min on a plate reader (FilterMax F3, Molecular Devices). Ten microliters of opsonized bacteria, which were incubated with 20% normal human serum (NHS) or heat-inactivated human serum in order to inactivate complement activity for 30 min at room temperature, was inoculated into neutrophils to a multiplicity of infection (MOI) of 10, and subsequently, the ROS production (relative luminescence unit) was collected kinetically every 2 min for up to 2 h on the plate reader. If required, the serum was treated with Futhan (BD Biosciences, #552035) before opsonizing bacteria to inhibit all complement pathways.

### Gentamicin protection assay

To enumerate *Salmonella* survival in neutrophils over the course of incubation, 5 × 10^5^ primary murine neutrophils or differentiated HL-60 cells were infected with 5 × 10^6^ opsonized *S*. Typhimurium (MOI = 10) in a 24-well plate. The plate was centrifuged at 250 × *g* for 5 min and incubated in an incubator (37°C, 5% CO_2_) for 30 min to allow neutrophils to take up bacteria. After incubation, 100 µg/mL of gentamicin was added. At 30, 60, and 120 min after infection, neutrophils were washed with phosphate-buffered saline (PBS), lysed by incubation with pre-chilled water for 15 min and spread on LB plates for enumeration of colony-forming units (CFU). Viability was assessed via trypan blue exclusion assay at 30 min and 2 h post-infection. After resuspending the pellet of the infected HL-60 cells in 1 mL of dPBS (Gibco, cat# 14190144), one part of 0.4% trypan blue (Gibco, cat# 15250061) was mixed with one part cell suspension (10 µL trypan blue: 10 µL cell suspension). Ten microliters was loaded onto each side of a dual-chamber cell counting slide (Bio-Rad, cat# 1450011), and trypan blue-positive (dead) and trypan blue-negative (live) cells were enumerated with the TC20 Automated Cell Counter (Bio-Rad, cat# 1450102).

### Measurement of C3 deposition on *S.* Typhimurium by flow cytometry

*S.* Typhimurium strains grown for 16 h in LB medium were opsonized by incubation with 1%, 5%, or 10% NHS or PBS as a mock control for 1 h at room temperature. After opsonization, bacteria were washed two times and fixed with sodium azide (final conc. 0.1%). Deposited C3 on the bacteria was stained by incubation with fluorescein isothiocyanate-conjugated antihuman complement C3 (MP Biomedicals, SKU: 0855167) for 1 h. Stained samples were analyzed by a flow cytometer (Cytek, Aurora), and cell populations were quantified using FlowJo software (Becton Dickinson and Company).

### *S.* Typhimurium infection of mice

For the systemic infection model, *S.* Typhimurium strains were grown overnight for 16 h, and a total of 2 × 10^4^ CFU of bacterial suspension in 0.1 mL PBS was inoculated via the IP route. Twenty-four hours after infection, all mice were euthanized, and their spleens, blood, and livers were collected at necropsy. Isolated spleens and livers were mechanically homogenized, and the resulting suspensions were serially diluted 10-fold and spread on LB plates supplemented with appropriate antibiotics to quantify bacterial colonization. One day after incubation at 37°C, colonies formed on each LB plate were counted to enumerate CFU. Significance of differences between experimental groups was determined by a one-tailed Mann-Whitney test using GraphPad Prism v.10. Statistical significance was set at *P* < 0.05.

### RT-qPCR assay

The expression of *pgtE* was quantified by RT-qPCR. *S.* Typhimurium strains were grown overnight, inoculated into fresh LB (Lennox) media, and incubated with aeration at 37°C for another 6 h. After incubation, bacteria harvested by centrifugation were treated with RNA Protect Bacteria Reagent (Qiagen, #1018380), and RNA was extracted using RNeasy Mini (Qiagen, #74104). Complementary DNA from each RNA sample was generated using reverse transcription PCR. Expression of *pgtE* and *hns* was determined using the ViiA7 Real-Time PCR System (Applied Biosystems) with primers DH54 (CGAACACTACATGCGCAAAC) and DH55 (ACCGCCCTTACCTTCTTCAT) for *the pgtE* transcript and qPCR_hns_f (TCTGAACAACATCCGTACTCTTC) and qPCRhns_r (TTCTTCTTCACGACGCTCATTA) for *the hns* transcript, as described previously (7) and SYBR Green PCR Master Mix (Applied Biosystems, #4309155). The obtained *pgtE* Ct value was normalized to the corresponding *hns* Ct value, and the fold changes of *pgtE* were determined. Differences between groups were determined using Student’s *t*-test on log-transformed data.

### Statistical analyses and software

All data are expressed as the mean ± standard error of the mean. GraphPad Prism v.10 was used for statistical analyses, and the test used to analyze each data set is indicated in the figure legend. Statistical significance was set at *P* < 0.05. The online tool Interactive Tree of Life was used to generate the tree shown in Fig. S2 (59).

## Data Availability

Data reported in the article figures are included in the supplement.

## SUPPLEMENTAL MATERIAL

Supplemental Figures mbio.01609-25-s0001.pdf Fig. S1 to S3.

Original data mbio.01609-25-s0002.xlsx Original data used to generate Fig. 1–6.

ASM does not own the copyrights to Supplemental Material that may be linked to, or accessed through, an article. The authors have granted ASM a non-exclusive, world-wide license to publish the Supplemental Material files. Please contact the corresponding author directly for reuse.
