# Supplementary material for: African Salmonella enterica serovar Typhimurium ST313 isolates prevent reactive oxygen species production by human neutrophils via elevated PgtE expression
Source: mBio. 2025 Jul 14;16(8):e01609-25. doi: 10.1128/mbio.01609-25 (PMC12345174; doi:10.1128/mbio.01609-25)
Supplement: Supplemental Figures — Fig. S1 to S3. [file mbio.01609-25-s0001.pdf]

Supplementary figures:

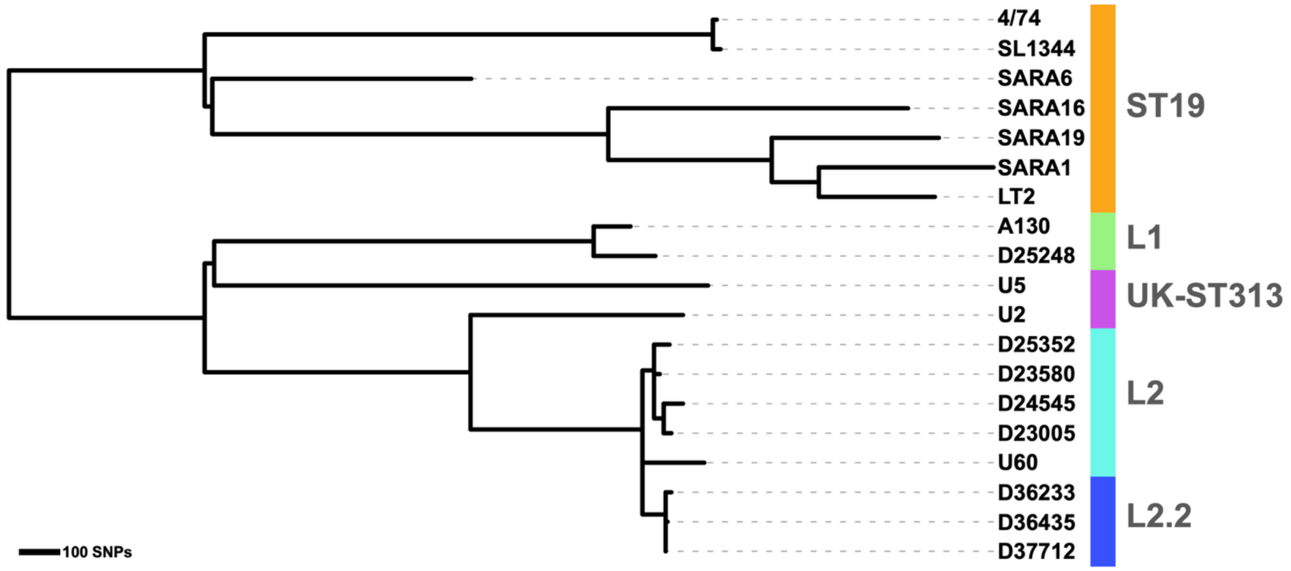

Figure S1, related to Fig. 4: Tree showing relatedness of sequence type (ST)19 and ST313 strains used for experiments in human (Fig. 4) and mouse (Fig. S3) neutrophils, made using iTOL v6. The different lineages of *S.* Typhimurium strains are shown at the right. L1, UK-ST313, L2 and L2.2 are lineages of ST313 assigned by comparison of sequenced bacterial genomes. Horizontal scale bar indicates a difference of 100 single nucleotide polymorphisms (SNPs) between genomes.

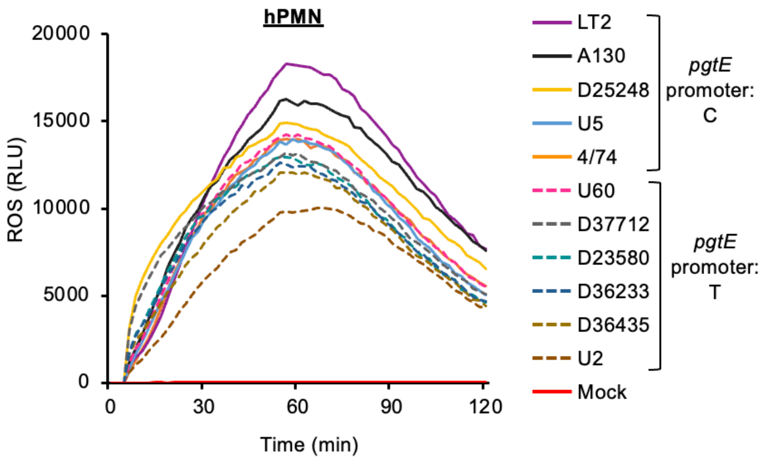

Fig. S2, related to Fig. 4: Representative experiment of five independent replicates comparing ROS elicited by *S.* Typhimurium strains carrying the *pgtE* C (solid lines) or T promoter variants (dashed lines) in primary human blood neutrophils (hPMN) inoculated at a MOI of 10:1 over a period of 120 minutes, as measured by chemiluminescence. RLU, relative luminescence units.

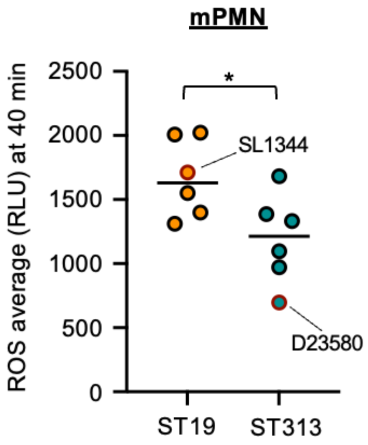

Fig. S3, related to Fig. 5: Mouse neutrophil ROS response to a panel of ST19 and ST313 strains**.**
ROS production elicited from murine bone marrow neutrophils (mPMN) inoculated at a MOI of 10:1 was compared between a group of ST19 (SARA1, SARA6, SARA16, SARA19, SL1344, LT2) and ST313 strains (A130 (L1), D26104 (L1), D25352 (L2), D24545 (L2), D23005 (L2), D23580 (L2)) by chemiluminescence. Differences between groups were determined using a Mann Whitney test. *, $p<0.05$. RLU, relative luminescence units; wt, wild-type
